# Control Software Design for a Multisensing Multicellular Microscale Gas Chromatography System

**DOI:** 10.3390/mi15010095

**Published:** 2023-12-31

**Authors:** Qu Xu, Xiangyu Zhao, Yutao Qin, Yogesh B. Gianchandani

**Affiliations:** 1 Center for Wireless Integrated MicroSensing and Systems (WIMS^2^), University of Michigan, Ann Arbor, MI 48109, USA; quxu@umich.edu (Q.X.); zxiangyu@umich.edu (X.Z.); 2 Department of Integrative Systems + Design, University of Michigan, Ann Arbor, MI 48109, USA; 3 Department of Electrical Engineering and Computer Science, University of Michigan, Ann Arbor, MI 48109, USA

**Keywords:** portable GC, embedded systems, C#, GUI, Python, I2C, SMbus, GPIO, JSON, firmware, middleware

## Abstract

Microscale gas chromatography (μGC) systems are miniaturized instruments that typically incorporate one or several microfabricated fluidic elements; such systems are generally well suited for the automated sampling and analysis of gas-phase chemicals. Advanced μGC systems may incorporate more than 15 elements and operate these elements in different coordinated sequences to execute complex operations. In particular, the control software must manage the sampling and analysis operations of the μGC system in a time-sensitive manner; while operating multiple control loops, it must also manage error conditions, data acquisition, and user interactions when necessary. To address these challenges, this work describes the investigation of multithreaded control software and its evaluation with a representative μGC system. The μGC system is based on a progressive cellular architecture that uses multiple μGC cells to efficiently broaden the range of chemical analytes, with each cell incorporating multiple detectors. Implemented in Python language version 3.7.3 and executed by an embedded single-board computer, the control software enables the concurrent control of heaters, pumps, and valves while also gathering data from thermistors, pressure sensors, capacitive detectors, and photoionization detectors. A graphical user interface (UI) that operates on a laptop provides visualization of control parameters in real time. In experimental evaluations, the control software provided successful operation and readout for all the components, including eight sets of thermistors and heaters that form temperature feedback loops, two sets of pressure sensors and tunable gas pumps that form pressure head feedback loops, six capacitive detectors, three photoionization detectors, six valves, and an additional fixed-flow gas pump. A typical run analyzing 18 chemicals is presented. Although the operating system does not guarantee real-time operation, the relative standard deviations of the control loop timings were <0.5%. The control software successfully supported >1000 μGC runs that analyzed various chemical mixtures.

## 1. Introduction

Microscale gas chromatography (μGC) systems have shown promise for use in in situ detection and measurement of volatile organic compounds (VOCs) since their introduction in the 1970s [1,2]. A μGC system incorporates a number of fluidic components for gas-phase flow management. The major fluidic components in μGC systems include preconcentrators, separation columns, detectors, pumps, and valves [3]. Typical operation involves two steps that constitute an analytical run: first, a sampling step is performed, in which the VOC molecules are drawn into the system using a sampling pump and adsorbed in the preconcentrator; subsequently, the VOC molecules are thermally desorbed and transported through the separation column by a carrier gas flow, during which the VOC molecules are spatiotemporally separated. Further downstream, the detector responds to the eluting analytes and forms a chromatogram, in which the location and magnitude of each peak indicate the species and quantity of a VOC, respectively.

The performance of μGC systems can be enhanced using complex architectures with a larger number of fluidic components. Some μGC systems incorporate multiple complementary detectors to enhance chemical recognition. These detectors are based either on a single sensing principle (e.g., capacitive detection [4], chemi-resistive detection [5], or resonance-based detection [6]) or on a mix of sensing principles with higher orthogonality [7]. A 2D μGC system typically incorporates two columns of complementary separation mechanisms to enhance the separation; typically, it requires either a thermal modulator [8] or a flow modulator [9]. The recently reported progressive cellular architecture [7,10] incorporates multiple sets of preconcentrators and columns, with each set tailored for a different range of VOC volatilities.

In a μGC system, the diverse fluidic components all require specialized controls. For example, the preconcentrators, separation columns, and detectors require separate temperature controls, as they are operated at different temperatures and with different heating rates. The heating control is usually provided by either pulse-width modulation (PWM) of a fixed voltage or by controlling an analog voltage. This control is preferably implemented in a servo-controlled (closed-loop) manner using temperature measurements from either thermistors or thermocouples connected to analog-to-digital converters (ADCs) [11,12]. Capacitive detectors are typically read out using capacitance-to-digital converters (CDCs) [4,7,10,13]; photoionization detectors are read out of amplifiers and ADCs [7,14]; resonance-based detectors are read out by phase-locked loop circuits [6] and ADCs. The valves used in μGC systems are typically active valves, either latching or non-latching. Latching valves are actuated using a short voltage pulse, the polarity of which controls whether the valve is opened or closed. In contrast, non-latching valves need sustained voltage levels but with an additional complexity favored for certain models, in which the sustained voltage should be lower than the initial actuating voltage. The commonly used miniature piezoelectric or motor-driven diaphragm pumps are controlled by on/off power and sometimes in combination with a tunable actuation frequency.

Within an analytical run, all the μGC components must be continuously and concurrently controlled. One of the challenges is that the data rates of different elements can vary widely. For example, the data rates of detectors vary based on the sensing principles and the readout electronics; higher data rates are generally favored. Other components may differ even more widely in data rates. The control software must accommodate the heterogeneous timing of individual components, with some components operating in closed-loop control.

The control software must also implement fail-safe protocols to handle hardware error cases, such as accidental overheating and thermal runaway. Otherwise, these error cases may cause irreversible damage to the μGC system.

To achieve standalone operation, all these hardware controls must be provided by an embedded computer (EC). Conversely, to facilitate user interaction, a user interface is often preferred on an external computer that can communicate with the EC.

This work presents advances in control software using a representative µGC system that is based on a multisensing progressive cellular architecture (MPCA) [7]. This MPCA system is an example of a highly complex μGC system with a large number and variety of components that require concurrent software control. The MPCA system incorporates eight elements that require individual temperature control, six detectors that require capacitive readout, three detectors that require voltage readout, six latching valves, and three pumps with different control requirements. This work investigates a multithreaded software architecture for the concurrent control of the MPCA system, with specific considerations regarding thread implementation, thread timing, temperature and flow control, communications between the EC and electronics, data saving and storage, low-power operation, and the user interface (UI) that communicates with the EC.

## 2. Design and Implementation

### 2.1. MPCA System Overview

**Figure 1:**
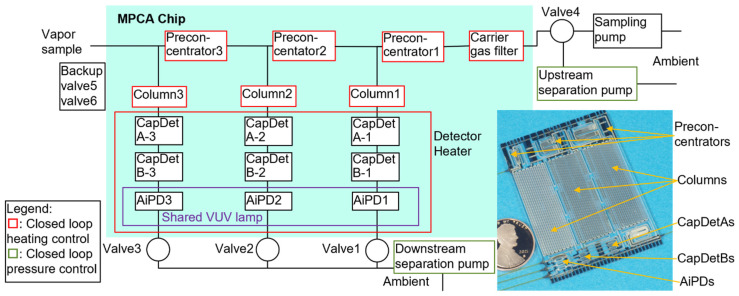
MPCA fluidic architecture and photo of the chip.

The MPCA system incorporates a monolithic microfabricated MPCA chip in addition to multiple miniature valves, pumps, and pressure sensors. The MPCA chip integrates three μGC cells, each containing a preconcentrator, a separation column, and three complementary detectors (Figure 1). The three cells are tailored to different ranges of analyte volatility, primarily by altering the sorbent materials in the preconcentrators and adjusting the stationary phase thicknesses in the separation columns. The three detectors within each cell are based on different sensing principles, providing complementary responses that enhance chemical recognition. Two of the detectors are capacitive detectors (CapDets) that use coplanar interdigitated thin-film metal electrodes with a sensing polymer of controlled thickness. The absorption of incoming analytes causes changes in both the thickness and dielectric constant of this layer, leading to changes in the detector capacitance. The first capacitive detector (CapDetA) primarily responds to polymer swelling, which produces only positive changes to the capacitance for all analytes. In contrast, the response of the second capacitive detector (CapDetB) is dominated by the change in dielectric constant, which may produce positive or negative changes to the capacitance depending on the dielectric constant of the analytes relative to that of the polymer itself. The third detector is an arrayed integrated photoionization detector (AiPD), which uses biased coplanar electrodes to draw a current from the photoionization of the analyte molecules. The photoionization is caused by ultraviolet radiation. The AiPDs across the three cells share a vacuum ultraviolet (VUV) lamp. Additionally, the MPCA chip incorporates a carrier gas filter, which dehumidifies ambient air to supply the carrier gas. For each preconcentrator, each separation column, the carrier gas filter, and the ensemble of all the detectors, a pair of on-chip heater and thermistor are incorporated to support closed-loop temperature control.

The MPCA system operation includes a sampling step, three sequential separation steps (one for each cell), and multiple purging steps. During the sampling step, the flow is generated using a sampling pump and routed using the valves. The analyte samples are transported through the preconcentrators sequentially, first through the one with the least adsorptive sorbent and then through those with progressively more adsorptive sorbents. The analytes are adsorbed by the preconcentrators, with the lower-volatility chemicals adsorbed by the upstream preconcentrator, and the more volatile chemicals breaking through the upstream preconcentrator and being adsorbed by downstream preconcentrators. During the separation step for each cell, the corresponding preconcentrator is heated to desorb the chemicals, which are then carried forward by the carrier gas flow and separated by the corresponding column and subsequently detected by the detectors. The carrier gas flow during separation is generated using both an upstream separation pump and a downstream separation pump, both of which may be used in conjunction with a pressure sensor to support closed-loop flow control. Prior to the next sampling step, purging steps may be used to reduce humidity and chemical carryover; these purging steps involve combinations of component heating and carrier gas flow. To support the MPCA system operation and continuing development, the control software must provide users with enough flexibility to modify operation sequences and parameters. 

### 2.2. Electronic Interfaces 

**Figure 2:**
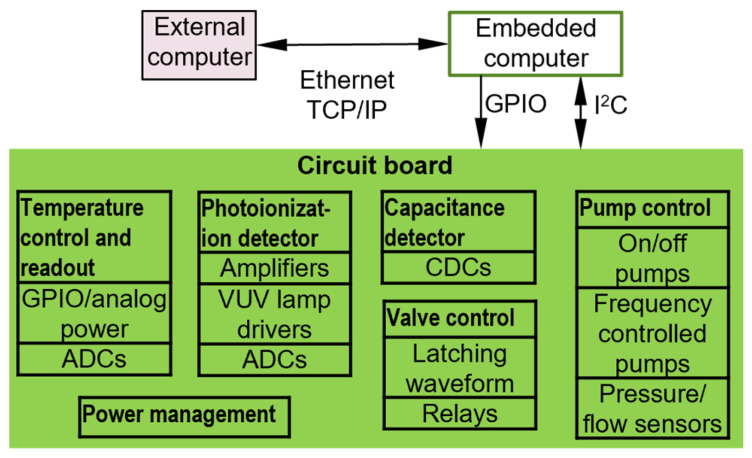
Block diagram of the electronic interface between components.

The EC serves as both the controller for the MPCA system electronics and the interface with the laptop UI (Figure 2). The EC may be embodied as either a microcontroller [11] or a single-board computer [5]. Microcontrollers are highly compact and inexpensive, but their limitations in computational power, visualization, and storage resources increase the burden on software development. In contrast, single-board computers provide a favorable balance between device size, cost, computational power, and ease of software development. The control and readout functions in this work can be supported by either a microcontroller or a single-board computer. However, from the perspective of a fully autonomous system for continuous, near-real-time in situ analysis, the post-processing of raw data requires extensive computation [15,16]. An alternative to the EC is a data acquisition (DAQ) device [17]; it can be easily programmed and is attractive for proof of concept. However, without a built-in controller, the control software for a DAQ device typically resides on an external computer, which does not always facilitate standalone operation. This work uses a Raspberry Pi (RPi) series single-board computer [18] as the EC. The RPi series includes various models with different computational power, power consumption, and sizes. Most models have the same pinout and the same operating system, which facilitates upgrades to newer models. For this work, the control software is built in the RPi Model 3B+ [18], which was the latest model available at the start of this work. 

The electronic interfaces used within the MPCA system are selected for their compatibility with the general-purpose input/output (GPIO) pins and/or the inter-integrated circuit (I^2^C), which are readily available on the EC. A GPIO pin can be easily programmed to provide simple on/off control or to provide square waveforms with controllable frequency and duty cycles. An I^2^C bus, operating with just two wires, may be used to communicate with tens of integrated circuit (IC) chips. This capability makes the I^2^C more suitable for the MPCA system than other protocols, such as the serial peripheral interface (SPI) and the universal asynchronous receiver/transmitter (UART). 

**Figure 3:**
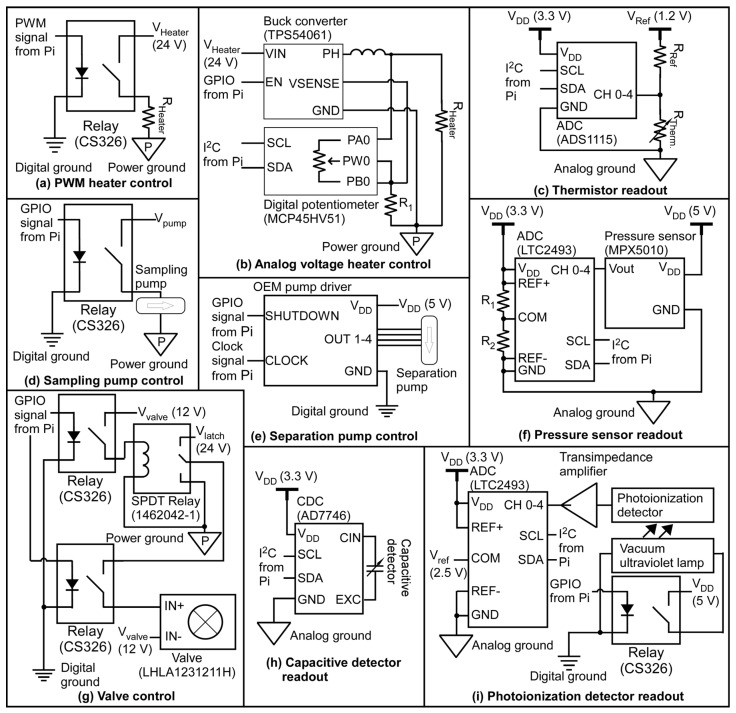
Circuit schematics of μGC system hardware.

The component heating is controlled by pulse-width modulation (PWM) for certain elements and by analog voltage control for other elements. The PWM-based circuit uses a relay (#CS326, Coto Technology, North Kingstown, RI, USA) controlled by a PWM signal from the EC (Figure 3a). By varying the duty cycle of a 24 V square wave, the PWM signal controls the average output voltage using just one GPIO pin, while eliminating additional ICs. However, for certain components that are sensitive to electrical interference, an analog voltage control circuit is desired. This is implemented using a digital potentiometer (# MCP45HVX1, Microchip Technology, Chandler, AZ, USA) that controls the output voltage of a buck converter (# TPS54061DRBR, Texas Instruments, Dallas, TX, USA) (Figure 3b). In the MPCA system, the heating of the preconcentrators, columns, and carrier gas filter uses the PWM-based circuit, whereas the heating of the ensemble of the detectors uses the analog voltage control circuit.

For temperature measurements, each on-chip thermistor is read out using a voltage divider coupled to an ADC (#ADS1115, Texas Instruments, Dallas, TX, USA) (Figure 3c). This ADC provides 16-bit resolution at a data rate of 8–860 Hz. It contains a converter core that is shared by four input channels in a time-multiplexed manner. Therefore, the eight on-chip temperature channels can be read out using two chips of this ADC, maintaining the same resolution but at ¼ of the data rate compared to an individual channel. These are necessary for the closed-loop temperature control of the MPCA system.

The sampling pump is a mini motor-driven pump (#NMP03KPDC-L, KNF Neuberger Inc., Trenton, NJ, USA) that is controlled using a relay (Figure 3d). The separation flow requires more accurate and adjustable control. The two separation pumps are piezoelectric micropumps (#mp6-air, Servoflo Corporation, Lexington, MA, USA), each driven using an original equipment manufacturer (OEM) driver circuit. The driver circuit receives on/off control from a GPIO pin; it also receives a square wave with a variable frequency of 0–1000 Hz to control the flow rate of the separation pump (Figure 3e). To implement closed-loop control of the separation flow rate, two differential pressure sensors (#MPX5010DP, NXP Semiconductors, Eindhoven, The Netherlands) are used to measure upstream and downstream pressure heads generated by the two separation pumps; these two pressure sensors are directly read out using a high-resolution ADC (#LTC2493, Linear Technology, Milpitas, CA, USA) (Figure 3f), which is rated with a 24-bit resolution at a data rate of 7.5 Hz. It contains a converter core shared by four time-multiplexed input channels. Therefore, the two pressure sensors can be read out using one chip of this ADC, maintaining the same resolution but at half of the data rate (i.e., 3.75 Hz) compared to an individual channel.

Six latching solenoid valves (#LHLA1231211H, The Lee Company, Westbrook, CT, USA) are used to route gas flow within the system. A single-pole double-throw relay (#1462042-1, TE Connectivity, Bevaix, Switzerland) is used to select the voltage level between that required for latching and unlatching a valve. Additionally, multiple relays (#CS326, Coto Technology, East Greenwich, RI, USA) are used to separately control the actuation of individual valves (Figure 3g).

Each of the two CapDet elements in the active cell is read out using a separate high-resolution capacitance-to-digital converter (CDC) (#AD7746ARUZ, Analog Devices Inc., Wilmington, MA, USA) (Figure 3h); the CDC is rated for a root mean square (RMS) input-equivalent noise level of 4 aF and a peak-to-peak noise of 27 aF, at a data rate of 9.1 Hz. A limitation of this CDC is its single fixed I^2^C address, which allows for only one CDC chip on each I^2^C bus. This limitation is overcome by using an I^2^C address translator (#LTC4316, Linear Technology, Milpitas, CA, USA) to assign different I^2^C addresses to the additional CDC chips. All the CDC chips are set in continuous conversion mode, operating at the slowest data rate (9.1 Hz) to achieve the highest available resolution. The excitation signal is set at 32 kHz frequency and ±1.65 V peak-to-peak amplitude. The built-in capacitance offset is set at 13.344 pF, which provides an effective measurement range of 9.248 pF to 17.440 pF. More details of the CDC chip connectivity are described in Supporting Information Section S1.

For the AiPDs, the shared VUV lamp is controlled using on/off power. The current generated by each AiPD is amplified and converted into a voltage signal using a low-noise transimpedance amplifier circuit. This voltage is read out using a high-resolution ADC (#LTC2493, Linear Technology, Milpitas, CA, USA), which is the same model as that for the pressure sensors (Figure 3i). Because of the sequential (i.e., non-concurrent) operation of the separation steps in the three cells, the three AiPDs can be read out using three channels of this ADC chip without time multiplexing. Therefore, each AiPD can be read out at a nominal data rate of 7.5 Hz (which is twice as fast as for the pressure sensors).

**Figure 4:**
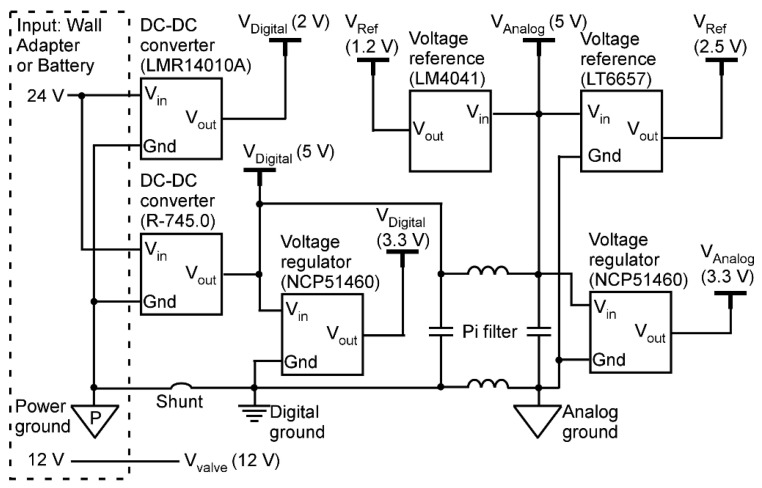
Schematic illustration of the power management circuit.

To power the MPCA system, both 24 V and 12 V supplies are needed, each of which can be sourced from either a wall plug power supply or a battery pack (Figure 4). The 12 V supply is dedicated to valve actuation. In contrast, the 24 V supply is used directly for component heaters (Figure 3a,b) and for valve actuation (Figure 3g). Additionally, the 24 V supply is used to provide 2 V for sampling pump actuation using a DC-DC converter (LMR14010A, Texas Instruments, Dallas, TX, USA). The 24 V supply is used to provide a 5 V supply, i.e., V_Digital_ (5 V), using another DC-DC converter (R-745.0, RECOM Power, Gmunden, Austria). The V_Digital_ (5 V) is used to provide a 3.3 V supply, i.e., V_Digital_ (3.3 V), using a voltage regulator (NCP51460, Onsemi, Scottsdale, AZ, USA). These digital voltage supplies are used for digital electronics. Additionally, the V_Digital_ (5 V) is treated using a Pi filter to provide another low-noise 5 V supply, i.e., V_Analog_ (5 V), which is used to provide another low-noise 3.3 V supply, i.e., V_Analog_ (3.3 V). These analog voltage supplies supply the data converters. The V_Analog_ (5 V) is also converted to 1.2 V and 2.5 V reference voltages for the ADC chips using two different power regulators (LM4041, Texas Instruments, Dallas, TX, USA and LT6657, Analog Devices Inc., Wilmington, MA, USA, respectively).

### 2.3. Control Software Implementation

In the MPCA system, the control software executed by the EC is programmed in Python 3.7.3. As the default language for the RPi, Python is compatible with a wide range of built-in and online open-source libraries, easing control software development. The main libraries that were used for this work include the open-source pigpio library [19] for GPIO pin control, the open-source SMbus library [20] for I^2^C communication, the Python built-in multithreading library for achieving multithreading, and the Python built-in JSON library for handling operation method files in JavaScript Object Notation (JSON) format. An operation method file contains all the necessary information for automating an analytical run. Each analytical run can be divided into up to eight run steps (such as a sampling step, a separation step, or a purging step). The operation methods file contains parameters for thermal, fluidic, detector, and timing controls of each run step. The libraries for interfacing with the electronics, namely SMbus and pigpio, are open-source libraries with core functions implemented in C programming language [19,20], which provides the necessary performance for the control software. The specific challenges and solutions are described in the following sub-sections.

#### 2.3.1. Multithreading Architecture

**Figure 5:**
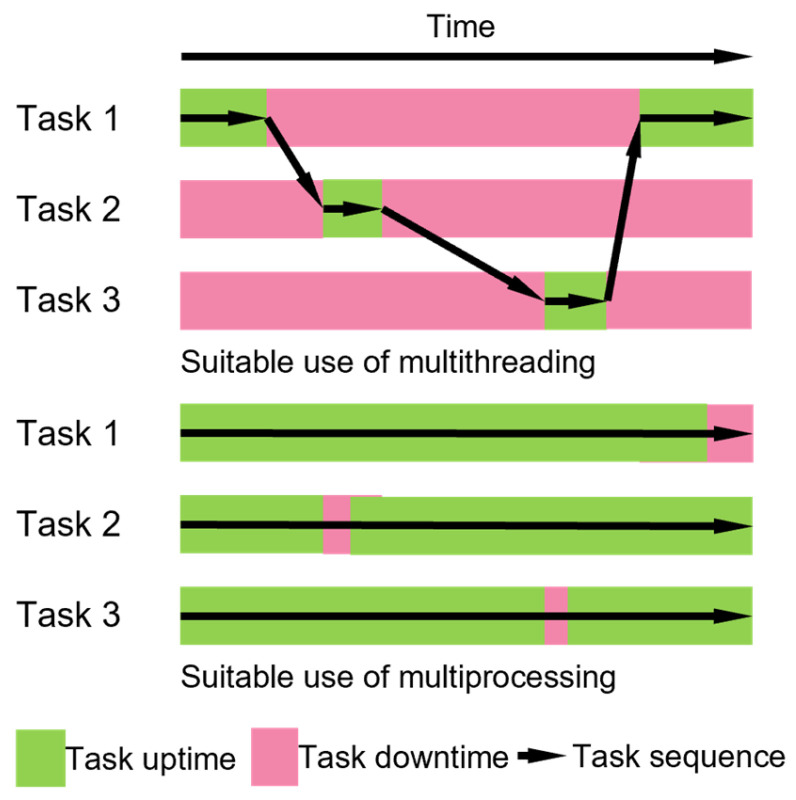
Temporal flow examples of multithreading and multiprocessing.

In each run step of the MPCA system operation, multiple fluidic components must be controlled concurrently. There are two mainstream approaches for providing concurrency: multiprocessing and multithreading (Figure 5). Multiprocessing provides true parallelism [21], where multiple tasks are executed simultaneously using multiple central processing unit (CPU) cores. It is most suitable for tasks that require intensive and parallel CPU usage. In contrast, multithreading achieves concurrency through context switching [22], where only one task is active at a time while the others are waiting. Multithreading is better suited for the MPCA system because all the readout ICs require relatively long data conversion intervals (of 15.6–146.9 ms) between measurements; increasing the computational power does not benefit the performance. In addition, multithreading is compatible with single-core CPUs that are commonly used in low-power single-core microprocessors (e.g., the RPi Model Zero [23]) that are favored for ultra-portable systems. Therefore, for this work, multithreading was chosen to provide concurrency.

**Figure 6:**
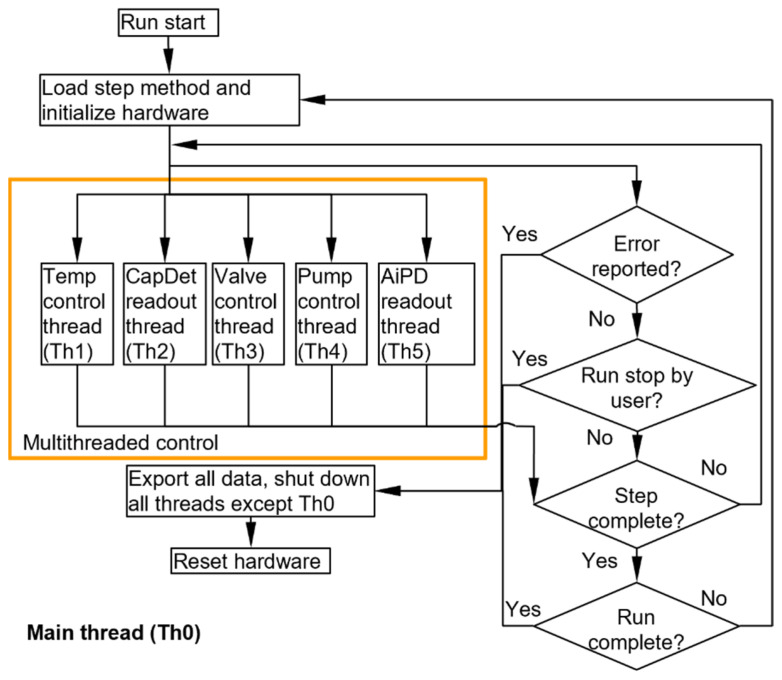
Flowchart of the main thread Th0.

To align multithreading with the MPCA system hardware, the control software is divided into six threads: the main thread (Th0), the temperature control thread (Th1), the capacitive detector readout thread (Th2), the valve control thread (Th3), the pump control thread (Th4), and the AiPD readout thread (Th5). Each thread operates using its own dedicated library that is specifically designed to interface with and control the corresponding electronics. The main thread manages the other threads, handles communication with the UI, monitors overall progress, saves readout data, and provides centralized control to start or stop all the threads as needed (Figure 6). 

#### 2.3.2. Concurrent Threads Th1–Th5

**Figure 7:**
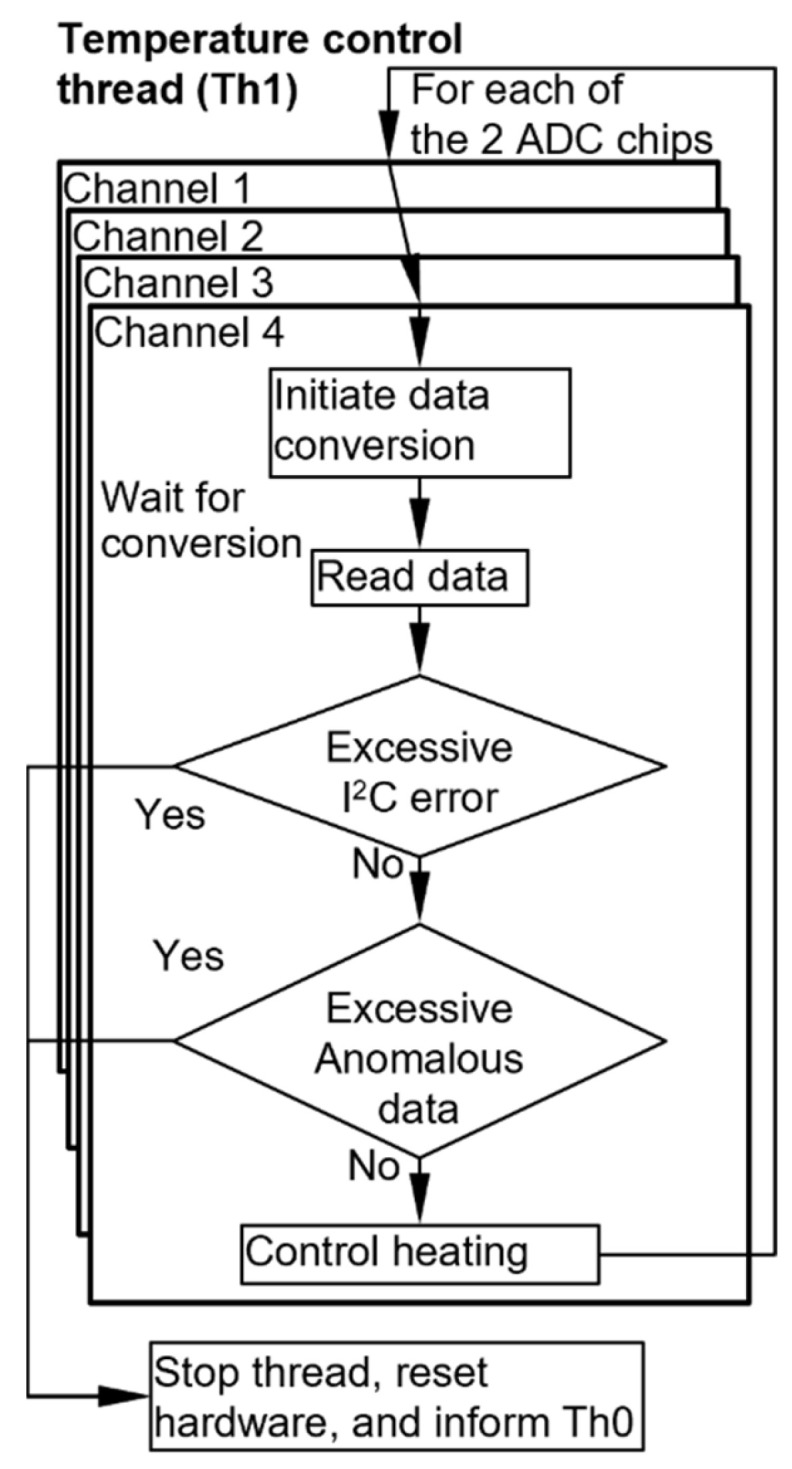
Flowchart of the temperature control thread.

The Th1 thread manages the closed-loop controls of all the eight temperature channels that are read using two ADC chips (Figure 7). In a control loop, it first initiates data conversion on one channel of each ADC chip and then enters sleep mode, awaiting the data conversion while allowing other threads to progress. Once the prescribed data conversion interval has elapsed, the Th1 thread reads the output data. If a channel is designated to perform active heating, its temperature data are used to compute the parameter that controls the heating power (as described in Section 2.3.4). The process is repeated for the four channels of each ADC chip, and the total duration of the four-channel process is recorded as the current loop execution time. The Th1 thread then re-enters sleep mode for a duration equal to the difference between the predetermined loop cycle time (which is 100.0 ms) and the execution time (which is typically between 62.5 ms and 100.0 ms). The Th1 thread also monitors any anomalous temperature readings, which indicate system malfunction that may lead to hardware damage if left uncorrected. In this process, for each temperature channel, if anomalous temperature readings occur consecutively over an excessive count (here set at three consecutive readings), this thread terminates all the heating and temperature readout actions; it also alerts the Th0 thread, which then terminates all the threads. 

**Figure 8:**
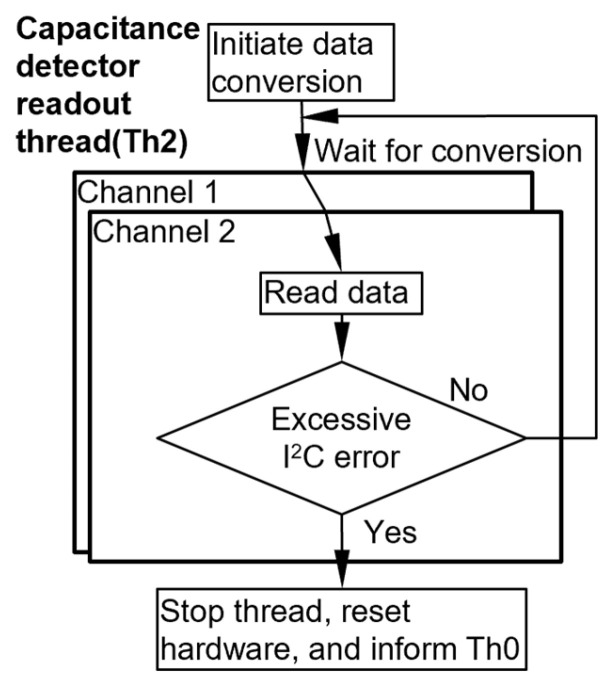
Flowchart of the capacitive detector readout thread.

The Th2 thread manages the readout of the two CapDet elements in each cell. The two CDC chips for CapDet readout are programmed to operate in the continuous data conversion mode, which automatically initiates a new data conversion immediately after the preceding one completes (Figure 8). After initiation, each CDC chip is read at a fixed interval, i.e., cycle time, of 110.0 ms, which is slightly longer than the conversion interval of 109.6 ms (Table 1). 

**Figure 9:**
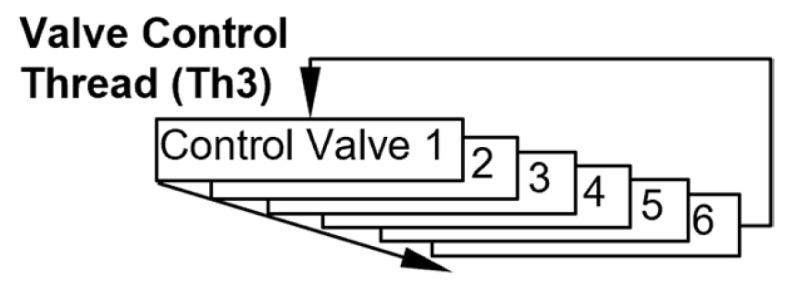
Flowchart of the valve control thread.

The Th3 thread controls the six latching valves (Figure 9). The valves are actuated sequentially using voltage pulses of 50.0 ms duration. The cycle time of Th3 is set at one second in order to reduce the computational burden (Table 1).

**Figure 10:**
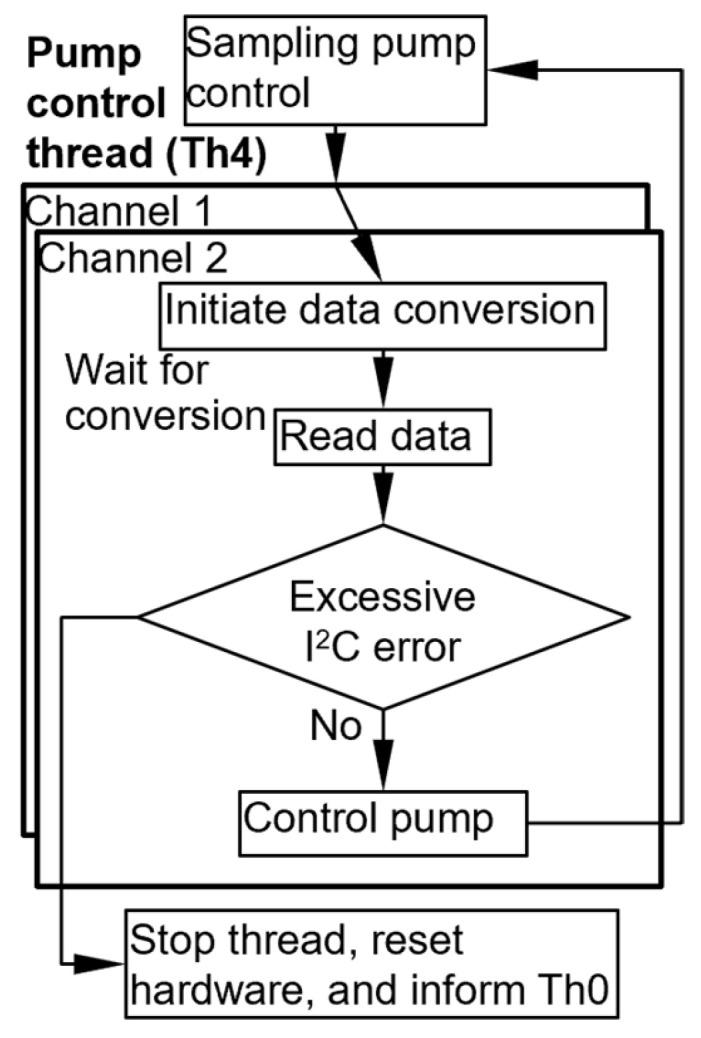
Flowchart of the pump control thread.

The Th4 thread controls the three pumps (Figure 10). For the sampling pump, the thread sends an on/off signal to start or stop the sampling pump. Then, for the separation pumps, it initiates data conversion for pressure head readout on one of the two ADC channels and then enters sleep mode. After the prescribed data conversion interval has elapsed, the Th4 thread reads the output pressure head from this channel and computes the actuation frequency required for its corresponding separation pump control (as described in Section 2.3.4). This process is then repeated for the other ADC channel. To accommodate the total required conversion time of both channels, which is 293.8 ms, the loop cycle time of the thread is set at 400.0 ms (Table 1). This is a much longer loop cycle time, leaving enough margin for any algorithmic processing that may potentially be required. 

**Figure 11:**
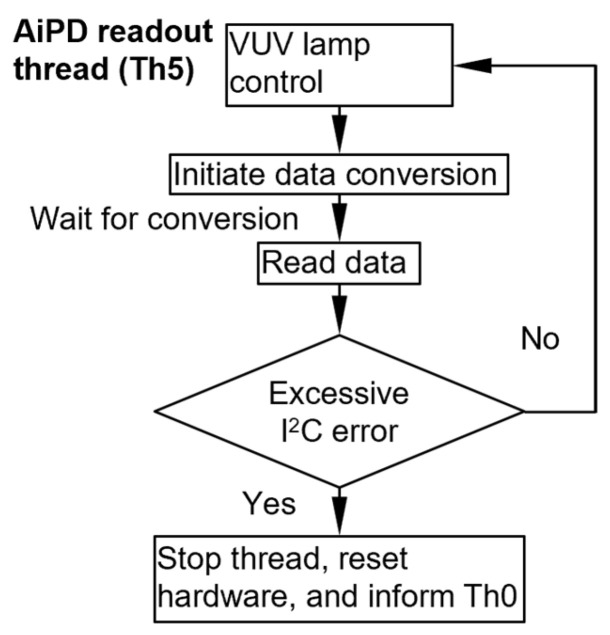
Flowchart of the AiPD output voltage readout thread.

The Th5 thread controls the AiPD power supply and readout of the AiPD (Figure 11). In its loop, it first sends an on/off signal to the VUV lamp. Next, it initiates data conversion on the ADC and then enters sleep mode. Ater the prescribed data conversion interval, the Th5 thread reads the output data. To accommodate the data conversion time of 146.9 ms, the cycle time of the thread is set at 200.0 ms (Table 1). Although this cycle time can be reduced, it is set at 200 ms for consistency with the Th4 thread, where the same ADC model is used but with two time-multiplexed input channels. 

Within each run step in the operation method, the Th2–Th5 threads are programmed to be active only when their specific functions are required. Otherwise, these threads enter sleep mode. For example, during the sampling step, where users typically do not need detector readouts, both the Th2 and Th5 threads enter sleep mode. When in sleep mode, a thread performs no action other than checking periodically—once per cycle time—to determine whether it needs to resume activity. Sleep mode conserves computational power and energy expenditure. 

#### 2.3.3. Thread Timing

The Raspberry Pi OS, which is the main operating system on RPi, is not a real-time operating system (RTOS). Consequently, programmed events are not always executed at precisely scheduled time points [24]. The timing errors can accumulate and consequently affect the accuracy of the chromatograms. To address this, two solutions are implemented. Firstly, the loop cycle time in each thread is set to be sufficiently longer than the time required by the hardware (Table 1), as described above. Secondly, all readout data are timestamped relative to the start of each run step. This allows back-end chromatogram processing (although not performed for this work) to interpolate the data, thereby compensating for any accumulated timing error, obtaining time-corrected chromatograms. 

#### 2.3.4. Control of Temperature and Flow 

In this work, all the temperature and pressure head channels are closed-loop controlled to follow user-defined transient temperature and pressure head profiles. After reading out the temperature or pressure head data, the corresponding output control is computed using a proportional–integral–derivative (PID) algorithm. For a parameter *x* under control, a unified transfer function of the control is as follows:

(1) $$C=C_{\max}(P\cdot \Delta x_{n}+I\cdot \Delta x_{\sum }+D\cdot (\Delta x_{n}-\Delta x_{n-1}))$$

where *C* is the control signal strength; *C*_max_ is the maximum available signal strength; *P*, *I*, *D* are the proportional, integral, and derivative coefficients, respectively; Δ*x_n_* is the difference between the target value of *x* and the measured value of *x*, both at the current (i.e., most recent) measurement time, n. The measurement time is discretized in 0.1 s intervals; Δ*x_∑_* is the accumulated difference between the target value of *x* and the measured value of *x* since the beginning of each run step; Δ*x_n_*_−1_ is the difference between the target value of *x* and measured value of *x*, both at the preceding (i.e., second most recent) measurement time. In temperature control, *x* represents the temperature. Further, for the PWM-based control, *C* represents the output duty cycle, with a maximum available value (i.e., *C*_max_) at 100%. For the analog voltage control via a potentiometer, *C* represents the output resistance, whereas *C*_max_ represents the maximum available output resistance of the potentiometer. In pressure head control, which effectively controls the flow rate, *C* represents the actuation frequency for a separation pump, *C*_max_ represents the maximum available actuation frequency, and *x* represents the pressure head.

The square waves required for PWM and for pump actuation may be generated using either hardware PWM or software PWM to provide the timing pulses. Hardware PWM supports precise timing control over a large range of frequency (up to 30 MHz) [18]. In contrast, software PWM is less precise in timing, because its pulses are generated using a software clock. Additionally, it can only support a lower frequency range (up to 40 kHz) [18]. Despite the superiority of the hardware PWM, on the RPi, it is limited to only 4 GPIO pins, which are insufficient for all the channels in the MPCA system. Considering the relatively large thermal time constants in the heating of the MPCA components (typically >0.5 s), all the temperature control channels are set to use software PWM. This software PWM operates at a frequency of 50 Hz with an 8-bit duty cycle resolution. Conversely, for the two separation pumps that require 0–1000 Hz frequency control, hardware PWM is selected. It provides a square wave at 50% duty cycle with a frequency control resolution down to a millionth of the set frequency. In this work, both types of PWM are managed by the pigpio library.

#### 2.3.5. Communication between the EC and the Electronics

As I^2^C bus is shared by the EC and multiple electronic components; the communication on this bus may be affected by power glitches, noise, and interference. However, I^2^C communications use a handshake protocol, so the Smbus used in this work can detect a failed handshake and report an error. If left unaddressed, accumulated I^2^C errors can prematurely end a thread without implementing proper hardware controls. This is especially risky during component heating, which may eventually damage the hardware irreversibly. Nevertheless, tolerating a limited number of I^2^C errors can sometimes be beneficial, especially at early stages of hardware development. Therefore, in the threads that use I^2^C, namely Th1, Th2, Th4, and Th5, an error-handling protocol is implemented as follows. Each thread continuously monitors its cumulative I^2^C errors. If the accumulated number of I^2^C errors in a thread exceeds a pre-determined threshold (here set at 3), the I^2^C communication is deemed unreliable. As a result, this thread terminates its associated hardware operation and alerts the main thread, which then shuts down all the other threads and saves the data. 

#### 2.3.6. Data Saving and Storage

The control software is designed to automatically export all raw data, including timestamps and values of the readout data points, into a comma-separated value (CSV) file at the end of each run step. CSV files are compatible with a wide range of commonly used software, such as Microsoft Excel, MATLAB, and Python. After an analytical run, the raw data files that are generated can be readily used for automated peak detection [16] and subsequently for automated chemical detection [15]. Additionally, after each run, the control software automatically saves as a metadata file, the operation method file that contains the entire set of operation parameters used for the run (which is further described in Section 2.3.8). This file also serves as a record of the exact operating parameters used for the run, which is important for systems where the operating parameters are frequently adjusted by users. For clear and convenient record keeping, all data and metadata files of a particular run are saved in a dedicated folder that is uniquely named with the hardware serial number and the date and time of the run. 

#### 2.3.7. Low-Power Mode

For applications with constrained power sources, e.g., small-size batteries or solar panels, μGC systems must operate within a limited power budget. Therefore, this work also investigates software approaches to reduce the power consumption of the MPCA system while maintaining essential hardware control capabilities. Table 2 shows various options for reducing power consumption by turning off certain RPi functions. Among these, the most significant power saving is achieved by turning off the Ethernet and USB functions. By implementing a low-power mode with all these options active, a total reduction in power consumption of 1.6–1.8 W is achievable. 

In the low-power mode, the MPCA system must be able to perform the analytical runs without Ethernet communication to the UI. Upon starting, the control software executes a script to terminate Ethernet and USB functions, then operates the MPCA system based on the previously transferred operation method file. After the prescribed runs are complete, the control software executes another script to restore Ethernet and USB functions, allowing users to configure the system for the next series of runs. Turning off the other RPi functions in the low-power mode does not affect the system function in any way.

#### 2.3.8. UI Design and Communications to the EC

**Figure 12:**
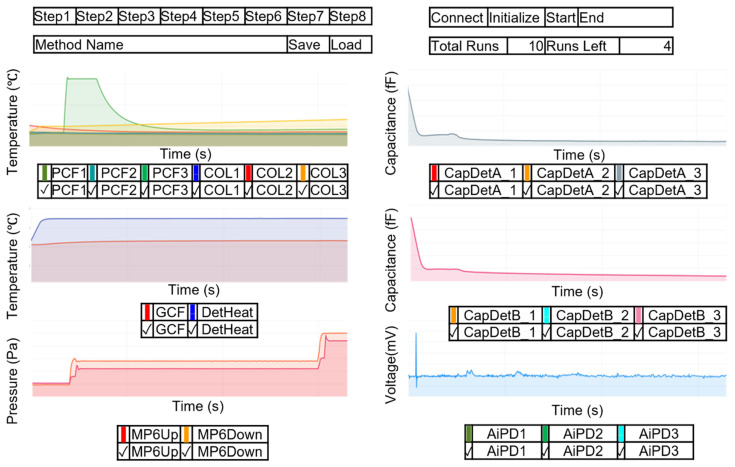
Graphical representation of the main UI window with real-time plotting of data.

The user interface (UI) of the MPCA system allows users to configure the operation method and to start and stop the system operation; it also provides a real-time display of the data. The UI operates in a Windows (Microsoft Corporation, Redmond, WA, USA) environment using the Windows Presentation Foundation (WPF) [25], which is a proven framework with strong open access library support. The UI is programmed in C# and Extensible Application Markup Language (xaml), which are the primary languages for WPF. In the UI, the main window contains plots for the temperature profiles, pressure head profiles, and the chromatograms. The plotting of real-time readout data (Figure 12) is implemented using LiveCharts [26]. To manage resource demands of plotting, each data stream is accompanied by a checkbox, which allows users to select whether to plot the data stream. After each run step, the plots are automatically cleared and prepared for the next run step. 

**Figure 13:**
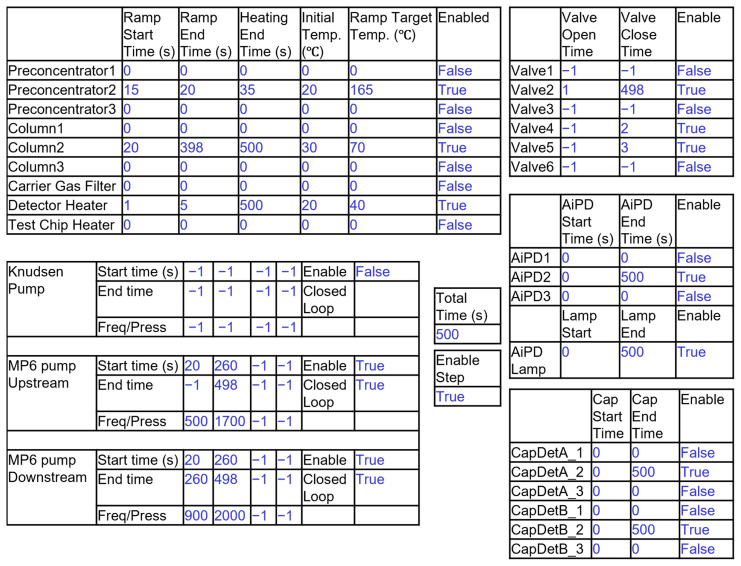
Graphical representation of UI for customizing the operation method. The entries in blue are representative values filled by a user for the Cell2 separation step.

The method configuration page allows the user to customize the operation parameters of each run step (Figure 13). This page opens when users click the corresponding button of a run step in the main UI window. The option to save the current method for future use is also included. More details about the UI are described in Supporting Information Section S2.

The operation parameters of all the run steps set up in the UI are arranged into an operation method file in the JSON format [27], which is both machine-readable and comprehensible to users. The widely used open-source Newtonsoft library is used to generate this file from the parameters entered into the UI. For the MPCA system, this file is typically larger than the TCP/IP receive buffer in the EC. Therefore, for transmitting it to the EC, this file is first segmented into smaller packets, which are then individually sent to the EC and finally reassembled into the full operation method file. The EC then reads the operation method file and prepares for initiating the MPCA system operation. During automated runs, the EC sequentially executes each run step based on the operation method file; in this process, the system operation is controlled by the EC and does not rely on the UI. The operation method file is also saved by the EC as the metadata for the corresponding MPCA system run.

In the normal operation mode, as opposed to low-power mode, real-time data transfer between the EC, and the UI is performed. This includes sending the readout data from the RPi to the UI and sending the user commands from the UI to the EC. For the readout data, the UI issues requests for the latest real-time data by sending a simple string to the EC. To avoid overburdening the UI, the frequency of these requests is programmed to vary inversely with the number of data streams already plotted. The EC then replies with the readout data in a string, which are parsed by the UI. The user commands include those to initialize the TCP/IP connection, to start the runs, and to stop the runs. These commands are sent to the EC in the form of a simple string immediately after being issued from the UI. 

## 3. Experimental Validation

The control software has been deployed in multiple generations and multiple units of the MPCA system [7] and has supported >1000 analytical runs, which have analyzed >40 different VOCs in various mixtures. Additional results of chemical analyses conducted using this control software have been previously reported [7]. The details of the system setup for experimental evaluation are described in Supporting Information Section S3. The performance of the control and readout can be assessed by the readout data from a typical run, as described below.

**Figure 14:**
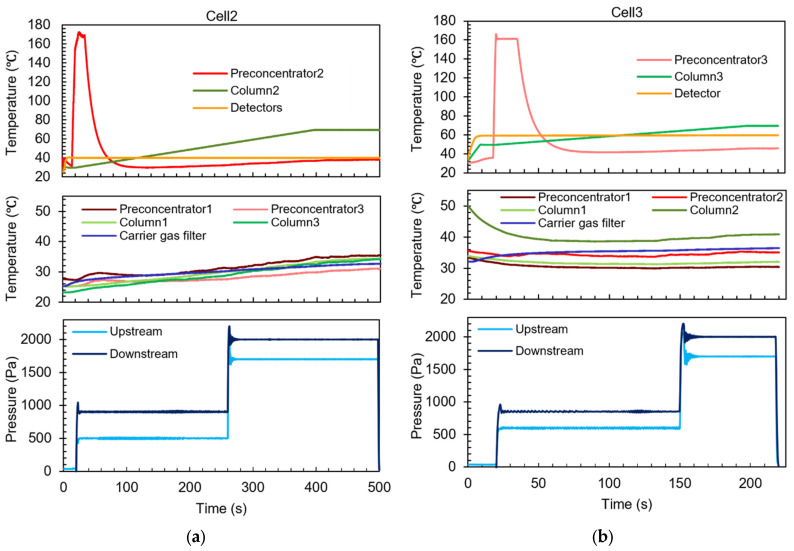
Typical temperature and flow control results in the MPCA system during (**a**) the Cell2 separation step and (**b**) the Cell3 separation step.

The efficacy of the closed-loop control was confirmed by verifying the measured temperature and pressure head profiles during the separation steps. For example, for the separation step performed in Cell2, Preconcentrator2 was set to ramp from 20 °C to 165 °C from 15 s to 20 s, to maintain a steady temperature at 165 °C from 20 to 35 s, and then to cool down naturally; Column2 was set to ramp from 20 °C to 70 °C from 20 s to 398 s and to maintain at 70 °C till 500 s; the detectors were set to heat up to 40 °C by 20 s and to maintain this temperature until 500 s. In a representative set of results, all these components adhered closely to their programmed temperature profiles (Figure 14a). Preconcentrator2 reached 93% of the target temperature at 20 s and remained within ±1% of the target temperature during the maintaining period. Column2 tightly followed its target temperature profile, showing an average deviation of only 0.38 °C. The region of the detectors also tightly followed its target temperature profile, showing an average deviation of only 0.02 °C. 

A parasitic temperature rise was observed in the unheated components, including Preconcentrator1, Preconcentrator3, Column1, Column3, and the carrier gas filter. This phenomenon was anticipated, because both the heated and unheated components were monolithically integrated in close proximity on the same MPCA chip, which had incomplete thermal isolation. Nevertheless, this parasitic temperature rise did not adversely affect the system operation. 

For the separation step performed in MPCA Cell2, each flow control loop, both the upstream and downstream, was programmed for a two-stage flow rate pattern: an initial modest flow rate, followed by a stronger flow. The initial flow was used to provide an amenable flow rate for separation, whereas the stronger flow was used to expedite the elution of the low-volatility chemicals in the cell. For example, the upstream loop was set to provide a pressure head at 500 Pa during 20–260 s and 1700 Pa during 260–500 s. From the pressure readout, the upstream pressure head stabilized by 30 s into a range of 485–513 Pa, which was within ±3.0% of the target pressure. Following the scheduled increase at 260 s, the upstream pressure head stabilized by 275 s within a range of 1704–1696 Pa, i.e., within ±0.3% of the target pressure. Similar control performance was also obtained for the downstream loop (Figure 12a).

For the separation step in Cell3, the operation parameters were set up in a similar manner as those for Cell2, albeit with slightly different values. Both heating and pressure head controls in Cell3 provided similar performance as Cell2 (Figure 14b). The unheated components also showed unremarkable temperature responses. For example, Column2 started at an elevated temperature because it was heated in the preceding separation step in Cell2; as it was not actively heated during the current separation step in Cell3, it gradually cooled down during this step. 

The effective control of the MPCA system was further validated by the chromatograms generated in analytical runs. In one such run, the MPCA system was tested with a mixture that included 18 detectable analyte chemicals, including cyclohexane, pentanal, heptane, pinacolyl alcohol, methyl isobutyl ketone (MIBK), toluene, butyl acetate, ethylbenzene, m-xylene, o-xylene, 1-chloropheptane, nitrobenzene, mesitylene, decane, 2-nonanone, undecane, and dodecane. All the chemicals were prepared at a concentration of 200 parts per billion (ppb), except for o-xylene, which was present at an unspecified concentration. The sampling time was set by the user to be 10 min. 

**Figure 15:**
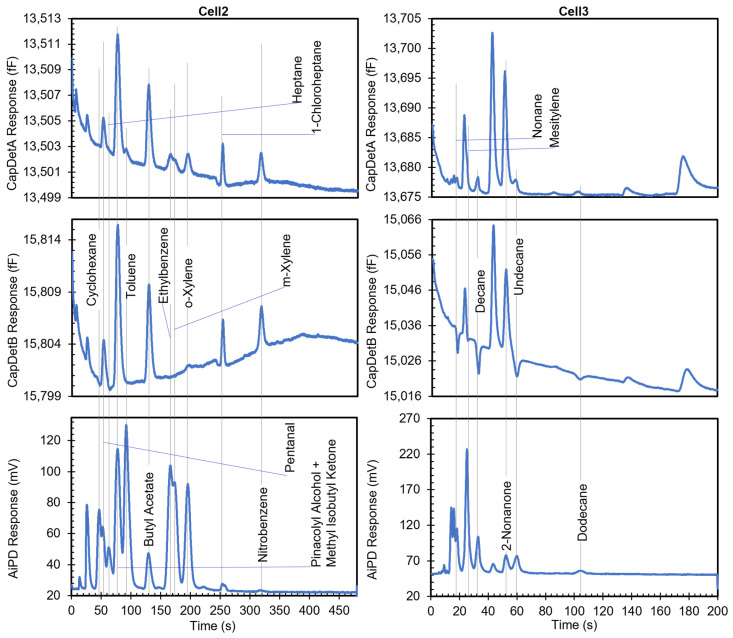
Chromatogram of a mixture containing cyclohexane, pentanal, heptane, pinacolyl alcohol, toluene, methyl isobutyl ketone, butyl acetate, ethylbenzene, m-xylene, o-xylene, 1-chloroheptane, nitrobenzene, nonane, mesitylene, decane, 2-nonanone, undecane and dodecane. Currently, the chromatograms are only presented for Cell2 and Cell3, as Cell1 is still under hardware development.

After the run, the exported data successfully provided an ensemble of chromatograms (Figure 15). The analyte chemicals presented various degrees of separation, such as pairs with complete baseline separation (e.g., between 1-chloroheptane and nitrobenzene), partial separation (e.g., between ethylbenzene and m-xylene), and complete overlap (e.g., between pinacolyl alcohol and MIBK). It was evident that the chromatograms obtained from the three different types of detectors were complementary in nature, even within each cell. For example, in Cell3, nonane produced relatively small positive CapDetA and AiPD peaks and a relatively large negative CapDetB peak, whereas ethylbenzene produced relatively small positive CapDetA peaks, almost no CapDetB peak, and a relatively large AiPD peak. 

Most peaks showed peak widths > 10 s, which were much larger than the data acquisition rate of any detector. Therefore, each peak was well profiled by at least 50 data points. Even for those peaks that were partially separated within a narrow time window (e.g., the three peaks in Cell3 from 17 s to 20 s), their profiles were well represented by >5 data points per peak. The available time resolution provided accurate and undistorted acquisition of the peak profiles, allowing recognition and quantification of the chemicals. 

**Figure 16:**
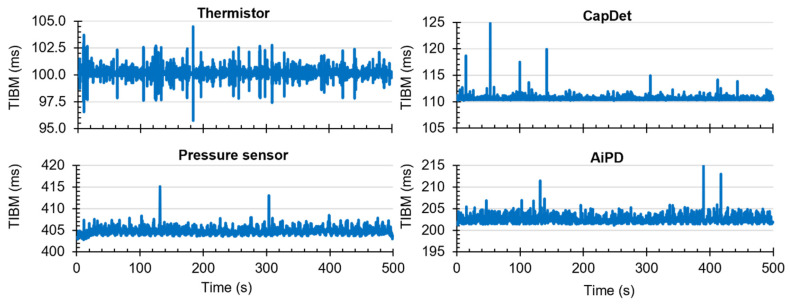
The typical experimentally measured time interval between measurement (TIBM) for each type of readout over time (measured during the MPCA Cell2 separation step). The number of data points in each plot is greater than 1200.

To examine the data acquisition rate more closely, readout data streams in the Cell2 separation step are used here as a case study. In this step, the data stream of each readout transducer type (i.e., temperature, CapDet, pressure head, or AiPD) contained >1200 data points. For each readout type, the actual time interval between measurements (TIBM) was obtained as the difference in the recorded timestamps between every two consecutive date points (Figure 16). Across all the readout types, the standard deviation of TIBM was <0.5% of the mean TIBM value, demonstrating timing stability (Table 3). This low variation in TIBM is desired, as it contributes to repeatable control of temperature and flow from run to run, which, in turn, contributes to repeatable system results. Additionally, the mean TIBM values for all readout types closely matched the intended cycle times, with <0.1% discrepancy in the temperature readout, <0.5% discrepancy in the CapDet readout, and <1.2% discrepancy in the pressure head and AiPD readouts. These discrepancies are further discussed in Section 4.

The noise level of each readout was also experimentally measured and compared to the rated noise in the converter datasheet (Table 4). The measured RMS noise values for the temperature, CapDet, pressure head, and AiPD readouts were 32.0 μV, 53.0 aF, 66.9 μV, and 107.1 μV, respectively. 

The measured noise of the temperature readout was comparable to that rated in the datasheet. According to the datasheet, the voltage resolution that corresponds to the least significant bit (LSB) of the ADC, under the settings in this work, is 62.5 μV, which aligns with the rated RMS noise. As shown by the measured noise waveform (Table 4), the measured voltage of the temperature readout typically toggled between two discretized values with an increment that corresponded to an LSB of the ADC. This observation not only confirmed the datasheet ratings but also indicated that the temperature readout resolution was limited by the LSB or the quantization error of the ADC.

In contrast, the CapDets measured much higher noise than that rated in the CDC datasheet. Similarly, the pressure head and AiPD readouts (both using the same ADC model) measured noise levels that significantly exceeded that rated in the datasheet (Table 4). These observations indicated that the resolutions of these readouts were not limited by the quantization errors of the ADC and CDC models. Instead, they appeared to be limited by the overall noise of the system, which possibly resulted from various sources, such as the noise in the reference voltage, noise in the power supply, interference, and the amplifier. Additionally, the elevated noise observed in the CDC readings was likely caused by the large baseline capacitances of the CapDets.

## 4. Discussion and Conclusions

Beyond the demonstrated functionalities, the control software may be improved in several ways. Firstly, ringing behaviors observed in the pressure head profiles likely resulted from the relatively long loop cycles (0.4 s) used for controlling the separation pumps. This problem can be mitigated by using an ADC model with a faster data rate.

Secondly, the recorded loop cycle times for pressure sensor and AiPD readouts were slower than the targets by 1% for all the data points. This was most likely because the control software assumed the computation times of these threads were negligible in the pressure sensor control thread (Th4) and the AiPD readout thread (Th5), whereas, in fact, the computation times of these threads were not negligible and caused some delay. In contrast, this issue was less pronounced in the temperature control thread, likely because of the implementation of a variable cycle wait time based on the measured computation time. In the capacitance readout thread Th2, the behavior of slower loop cycle time was also less significant, because the CDC chips operated in the continuous conversion mode, requiring only two I^2^C read operations per loop. For the pressure sensor control thread (Th4) and the AiPD readout thread (Th5), the approach of using variable cycle wait time can be implemented in the future. 

In conclusion, the experimental results demonstrate that the control software reported in this work is successful in using multithreading to provide concurrent control of all aspects of the μGC system operation, including temperature control, flow control, capacitive detector readout, AiPD readout, and user interface. The efficacy of the control software is reflected by the generated temperature profiles, pressure head profiles, and chromatograms, all aligning with the user-defined operation method and expectations. Despite the use of a non-real-time operating system, the variations in TIBM are small and show no adverse impact on system control. The raw chromatogram data acquired can be readily processed, first using an automated peak detection algorithm [16] and then using an automated chemical recognition algorithm [15], culminating in a fully automated VOC analyzer.

With the demonstrated functionality for the MPCA system, the control software reported in this work shows great potential for applications in other μGC systems. Its modular thread design can be easily expanded or adjusted to support different hardware configurations. In the future, real-time data transfer between the RPi and the UI can be implemented wirelessly, e.g., via Bluetooth or Wi-Fi. Additionally, the control software can be tested and assessed on a lower-power, single-core processor (e.g., the RPi Zero) to further reduce power consumption.

## Figures and Tables

**Table 1:** The IC model and conversion time used for each type of readout.

Readout Type	IC Model	OCCT (ms)	Time Multiplexed?	ECCT (ms)	PLCT (ms)
Temperature	ADS1115	15.6	Yes (4 channels)	62.4	100.0
CapDet	AD7746	109.6	No	109.6	110.0
Pressure head	LTC2493	146.9	Yes (2 channels)	293.8	400.0
AiPD	LTC2493	146.9	No	146.9	200.0

Abbreviations. OCCT: one channel conversion time; ECCT: effective channel conversion time (depending on whether multiple channels are time-multiplexed); PLCT: programmed loop cycle time.

**Table 2:** Normal-mode and low-power mode power consumption of the RPi (Model 3B+ in this work).

Functionalities	Normal Mode	Low Power Mode Savings
Bluetooth + Wi-Fi	On	Off, 0.2 W reduction
Soundboard	On	Off, 0.2 W reduction
Ethernet + USB	On	Off, 1.2 W reduction
HDMI	On	Off, 0.15 W reduction
Energy usage (idle state)	2.8–2.9 W	1.6–1.8 W reduction 1.1–1.2 W total consumption

**Table 3:** Actual time interval between measurements (TIBM) of all the readout component types (measured at the peak software workload step, i.e., the Cell2 separation step).

Readout Type	Intended Loop Cycle (ms)	Actual Mean TIBM (ms)	TIBM Standard Deviation (ms)
Temperature	100.0	100.1	0.4
CapDet	110.0	110.5	0.4
Pressure head	400.0	404.7	1.0
AiPD	200.0	202.3	0.9

**Table 4:** Noise data of the detectors.

Readout Type	Noise (RMS)		Typical Noise Waveform (over 5 s Duration)	
	Measured	Datasheet		
Temperature	32.0 μV (0.04 °C)	62.5 μV *	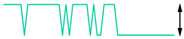	64.1 μV (0.85 °C)
CapDet	53.0 aF	4.0 aF	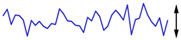	219.8 aF
Pressure head	66.9 μV (0.15 Pa)	0.6 μV *	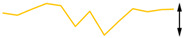	249.3 μV (0.55 Pa)
AiPD	107.1 μV	0.6 μV *	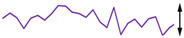	400.1 μV

* Per datasheet, this number is constant over a range of data rates and corresponds to the voltage for the least significant bit.

## Data Availability

The data presented in this study are available on request from the corresponding author.

## Supplementary Materials

The following supporting information can be downloaded at: https://www.mdpi.com/article/10.3390/mi15010095/s1, Section S1: Additional content on the CDC setup; Section S2: Additional contents on the graphical user interface; Section S3. Experimental Evaluation Setup.
