# Supplementary material for: Control Software Design for a Multisensing Multicellular Microscale Gas Chromatography System
Source: Micromachines (Basel). 2023 Dec 31;15(1):95. doi: 10.3390/mi15010095 (PMC10818470; doi:10.3390/mi15010095)
Supplement: Supplementary file 1 [file micromachines-15-00095-s001.zip › micromachines-2712018-supplementary.pdf]

Supporting Information

Control Software Design for a Multisensing Multicellular Microscale Gas Chromatography System

Qu Xu 1,2, Xiangyu Zhao 1,3, Yutao Qin 1,3,* and Yogesh B. Gianchandani 1,3,*

1 Center for Wireless Integrated MicroSensing and Systems (WIMS2), University of Michigan, Ann Arbor, MI 48109, USA; quxu@umich.edu (Q.X.); zxiangyu@umich.edu (X.Z.)
2 Department of Integrative Systems + Design, University of Michigan, Ann Arbor, MI 48109, USA
3 Department of Electrical Engineering and Computer Science, University of Michigan, Ann Arbor, MI 48109, USA
* Correspondence: yutaoqin@umich.edu (Y.Q.); yogesh@umich.edu (Y.B.G.)

S1. Additional content on the CDC setup

The CDC used in this work (#AD7746ARUZ) provides two time-multiplexed input channels; in principle, the 6 CapDets across the three μGC cells can be covered by three chips of this CDC (Fig. S1a). Because of the sequential (i.e., non-concurrent) operation of the separation steps in the three cells, the two CapDets in each cell are connected to two input channels that belong to two different chips of the CDC, hence avoiding time multiplexing in each CDC chip and allowing each CapDet to be read out at the maximum available rate for a given resolution. In this work, four CDC units are used to provide a level of design redundancy (Fig. S1b).

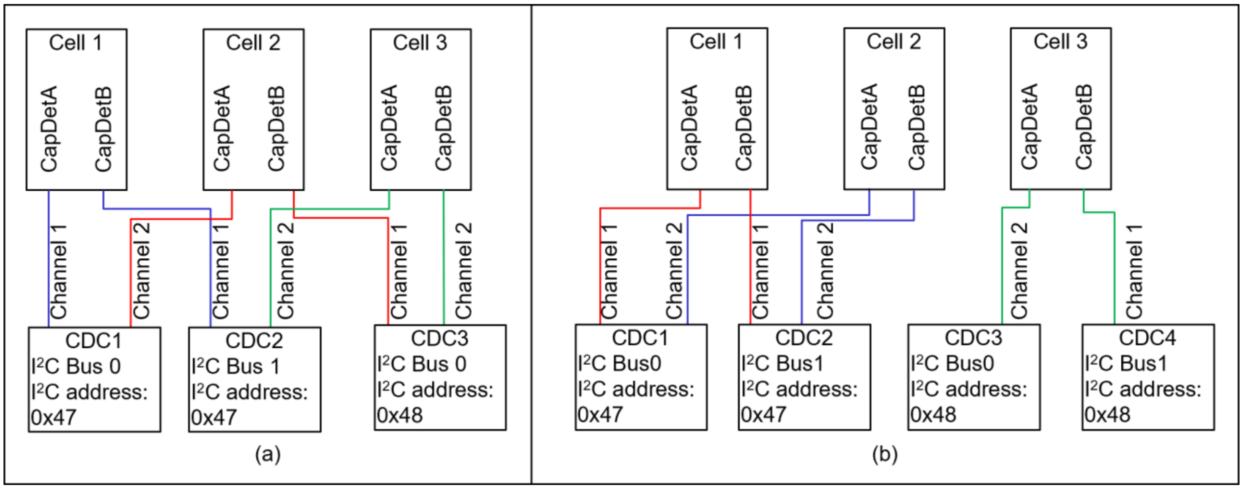

Figure S1. Illustration of connections between multiple CapDets and CDCs for the cases of (a) fewest CDC units needed, and (b) this work with a level of design redundancy.

S2. Additional contents on the graphical user interface

The graphical user interface (UI) provides the following functions: 1) facilitating the user in setting up run parameters; 2) saving and loading run parameters, 3) real-time plotting of data, and 4) issuing start and stop commands. In the main page (Fig. S2), the eight step configuration buttons "STEP1"-"STEP8" in the upper left area provides entries to the method configuration pages of up to eight individual steps that a single run may consist of. A textbox underneath these eight buttons is used to input the operation method file name. The "Save" and "Load" buttons

are used to save the currently set run parameters into the operation method file and to load a previously configured operation method file.

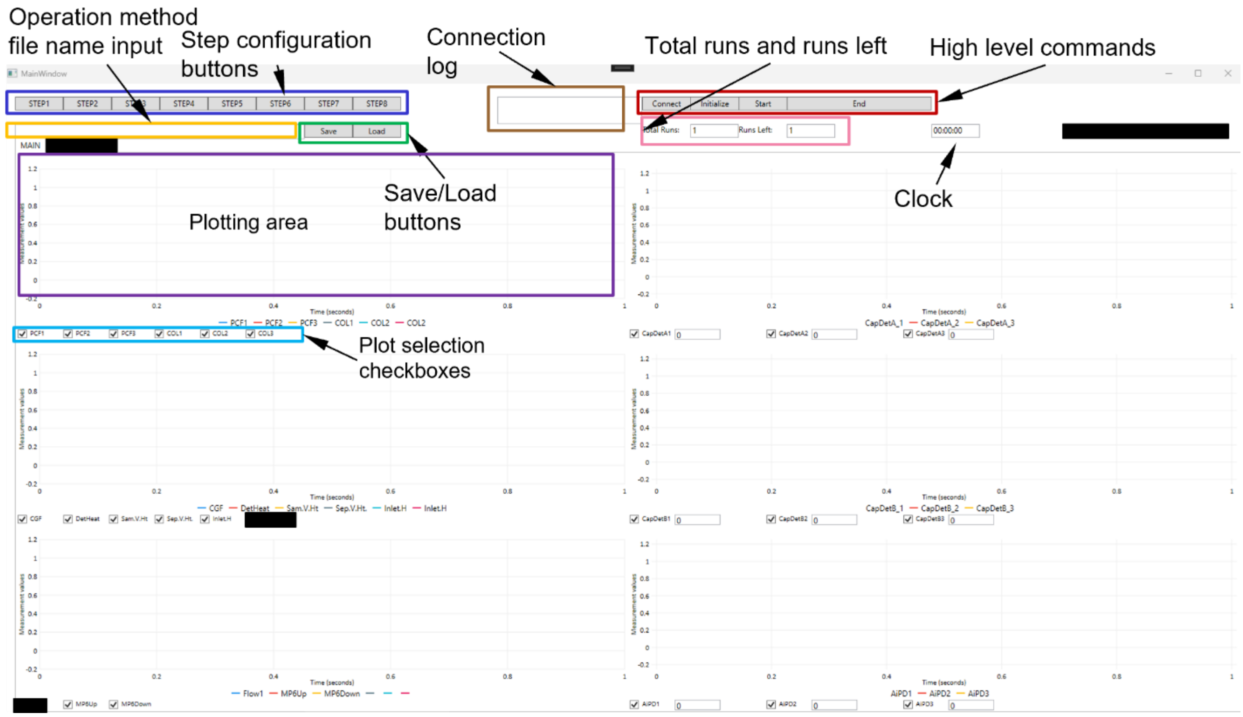

Figure S2. Main page of the UI. The obsolete or unrelated contents are covered by black boxes.

The four buttons in the upper center area of the UI main page (Fig. S2) are used to issue high-level commands to the embedded computer (EC). The “Connect” button is used to establish a TCP/IP connection with the EC. The result of the connection attempt (*i.e.*, successful or unsuccessful connection) is displayed in the textbox on the left side of the button. After the connection is established, pressing the “Initialize” button sends the run parameters (in the form of the operation method file) to the EC, which then automatically loads the run parameters for all the steps. Next, the “Start” button can be pressed to execute the run process, which is completely automated and controlled by the EC. The UI does not control the run automation process. During a run, if at any point the user needs to stop the run, the user can press the “End” button. Once the EC receives the “End” command, the EC terminates communication with the UI and saves all data.

If the EC encounters any unresolvable error, a message will be sent from the EC to the UI. A pop-up window will appear on the UI, notifying the user of the error. The EC saves all existing readout data, shuts down the MPCA hardware and stops communicating with the UI.

Underneath these four high-level control buttons is a “Total Runs” textbox, which is used to configure how many runs should be repeated. The neighboring “Runs Left” textbox is used to display how many runs are remaining. On the right side of the “Runs left” textbox is a clock showing the elapsed time since the beginning of the runs.

The six plots that constitute the majority of the UI main page are used to show the real-time readout data. The upper two plots in the left column are for temperature readouts; the bottom plot in the left column is for pressure readouts; the upper two plots in the right column are for capacitive detector readouts; the bottom plot in the right column is for AiPD readouts.

Underneath each plot are checkboxes to select or unselect which individual component(s) to plot. The checkboxes can be selected or unselected at any time during the run.

To set up a typical set of runs (Fig. S3), the user first starts the EC software and then the UI software. If a previously configured operation method needs to be modified, the user can click any of 8 step configuration buttons, which opens the step configuration window for the selected step (Fig. S4). After the user edits the parameters, the user can save the step parameters by clicking the “Save” button in this window, which then automatically closes and returns the user to the main window. After all steps are configured and the total number of repeated runs entered, the user can click the “Connect” button to connect with the EC, then click the “Initialize” button to load all the parameters, and finally click the “Start” button to start the automated control process on the EC side. The UI communicates regularly with the EC to fetch the most recent data. The step configuration buttons change to green color when the corresponding steps are completed or ongoing in a run and reset to grey after a run (Fig. S5).

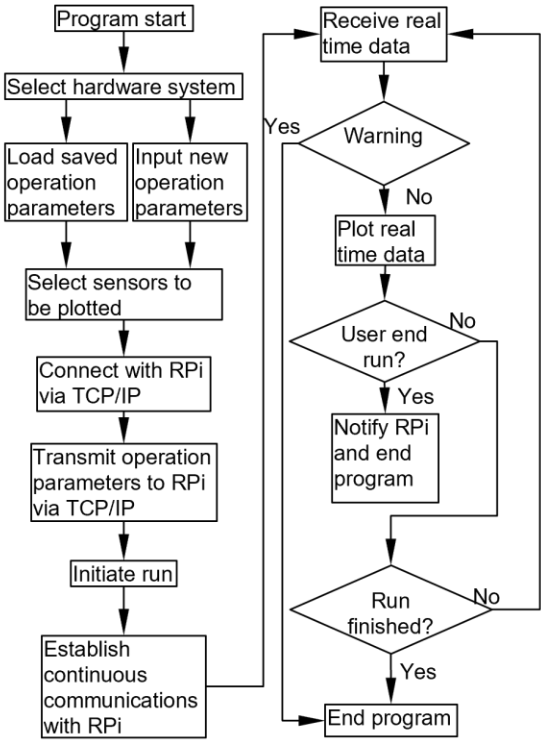

Figure S3. Flowchart of user interface operations.

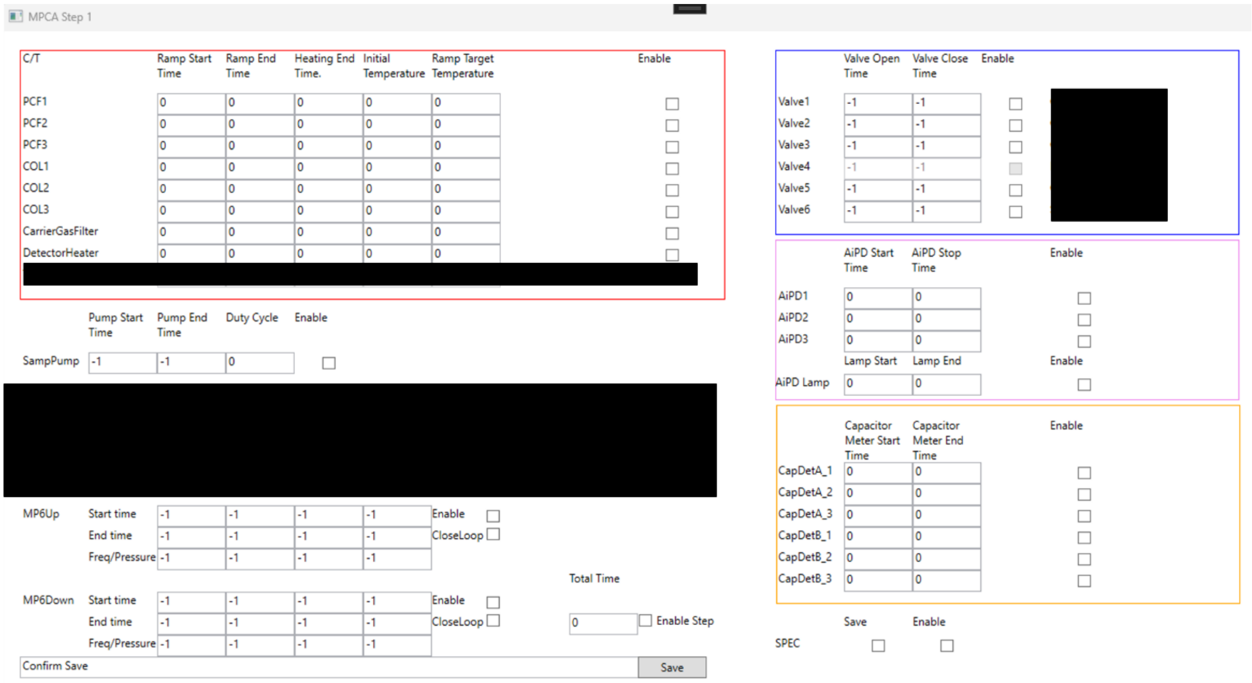

Figure S4. Method configuration page for editing the operation parameters of a run step. (The obsolete or unrelated contents are covered by black boxes)

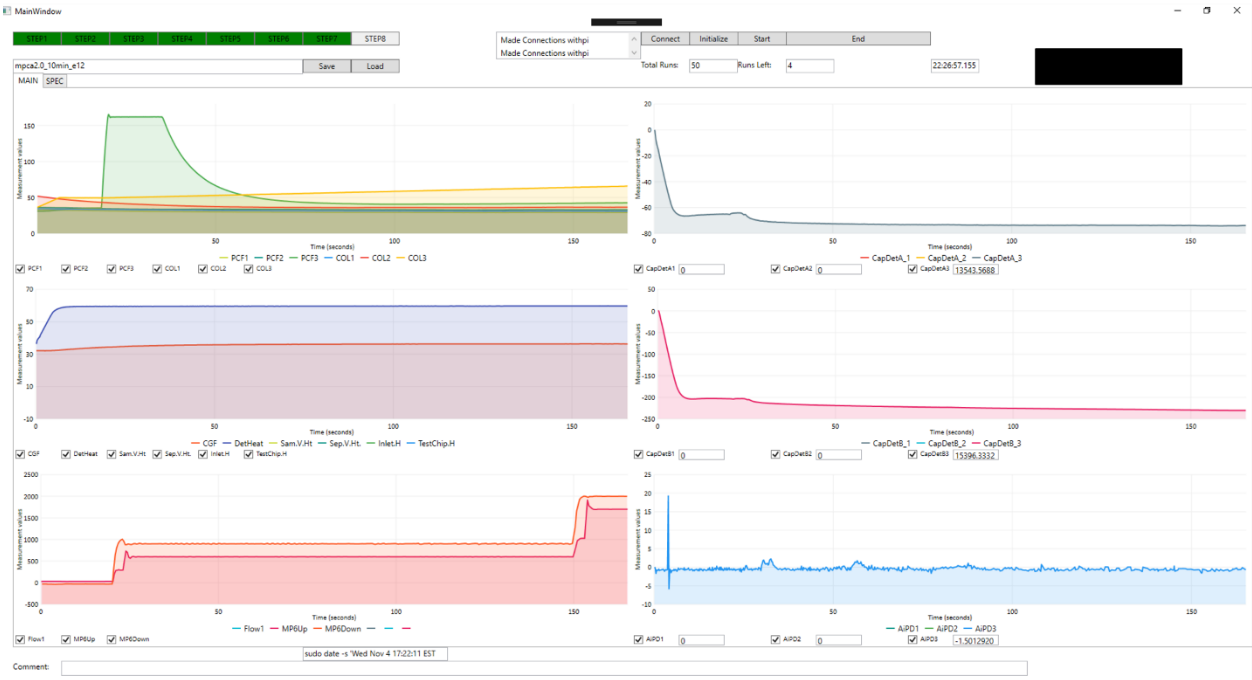

Figure S5. Main window during operation with real-time plotting of the readouts.

S3. Experimental Evaluation Setup

In a typical run that generated the results in the main paper, the operation method was configured with 7 steps. In the first step, the preconcentrators were purged by heating to 165°C for 30 to 40 s while actuating the upstream separation pump to provide the purge flow. Valve1,

Valve2, and Valve3 were all closed, whereas Valve4 was switched to the path of the upstream separation pump.

The second step was the sampling step, during which the sampling pump was turned on to provide the sampling flow through the preconcentrators, in the order of Preconcentrator1 (PCF1), Preconcentrator2 (PCF2), and preconcentrator3 (PCF3). In the final 45 s of the step, the carrier gas filter (CGF) was heated to 160 °C to be regenerated. PCF1 was also heated to 45°C to desorb water while also preserving the analyte chemicals. In this step, Valve1, Valve2, and Valve3 all remained closed, whereas Valve4 was switched to the path of the sampling pump. The sampling time was set to 10 minutes in this work.

In the third step, ambient air dehumidified by the CGF was pumped by both separation pumps operated together through the cells to purge out residual water vapor. PCF1 was heated to 45°C to continually reject water adsorption. Valve4 was switched to the path of the upstream separation pump, whereas Valve1, Valve2, and Valve3 were closed for purging the preconcentrators and opened for purging the separation columns.

The fourth step was the separation step of Cell1. Valve1 was opened, whereas Valve2 and Valve3 remained closed. PCF1 was heated to 165°C and maintained for 15 s to desorb the chemicals. The Cell1 column (COL1) was maintained at 25°C for isothermal separation. The separation flow was provided by both the upstream and downstream separation pumps. The capacitive detectors and photoionization detectors were turned on for active measurement.

The fifth step was the separation step of Cell2. Valve2 was opened, whereas Valve1 and Valve3 were closed. PCF2 was heated to 165°C and maintained for 15 s to desorb the chemicals. The Cell2 column (COL2) was linearly ramped from 30°C to 70°C from 20 s to 398 to provide temperature-programmed separation. The separation flow was provided by both the upstream and downstream separation pumps, targeting 0.6 sccm from 20 s to 260 s and 1.3 sccm afterwards. The capacitive detectors and photoionization detectors were turned on for active measurement and were maintained at 40°C.

The sixth step was an empty step set by the user as a backup for potential further treatment after the fifth step. The seventh step was the separation step of Cell3. Valve3 was opened, whereas Valve1 and Valve2 were closed. PCF3 was heated to 165°C and maintained for 15 s to desorb the chemicals. The Cell3 column (COL3) was linearly ramped from 50°C to 70°C to provide temperature-programmed separation. The separation flow was provided by both the upstream and downstream separation pumps, targeting 0.6 sccm from 20 s to 150 s and 1.3 sccm afterwards. The capacitive detectors and photoionization detectors were turned on for active measurement and were maintained at 60°C. The eighth step was an empty step as a backup for potential further treatment after the seventh step.
